# "Comparison of thyroid volume, TSH, free t4 and the prevalence of thyroid nodules in obese and non-obese subjects and correlation of these parameters with insulin resistance status"

**DOI:** 10.22088/cjim.11.3.278

**Published:** 2020-05

**Authors:** Parvin Layegh, Abbas Asadi, Ali Jangjoo, Parvaneh Layegh, Mohsen Nematy, Maryam Salehi, Aliakbar Shamsian, Golnaz Ranjbar

**Affiliations:** 1 **Endocrinology and Metabolism Metabolic Syndrome Research Center, Mashhad University of Medical Sciences, Mashhad, Iran **; 2 **Montaserie Organ Transplantation Hospital, Mashhad University of Medical Sciences, Mashhad, Iran**; 3 **Surgical Oncology Research Center, Mashhad University of Medical Sciences, Mashhad, Iran**; 4 **Department of Radiology, Faculty of Medicine, Mashhad University of Medical Sciences, Mashhad , Iran**; 5 **Clinical Nutrition Metabolic Syndrome Research Center , Mashhad University of Medical Sciences , Mashhad , Iran**; 6 **Social Medicine Clinical Research Unit, Faculty of Medicine, Mashhad University of Medical Sciences , Mashhad , Iran**; 7 **Department of Parasitology and Mycology, Faculty of Medicine, Mashhad University of Medical Sciences, Mashhad, Iran**; 8 **Department of Nutrition, Faculty of Medicine, Mashhad University of Medical Sciences, Mashhad, Iran**

**Keywords:** Obesity, Thyroid volume, Insulin resistance, Nodule, Function tests

## Abstract

**Background:**

To compare thyroid volume, thyroid stimulating hormone (TSH), free t4 and the prevalence of thyroid nodules between obese and non-obese subjects. Also, the association between BMI and insulin resistance status with various parameters of thyroid gland was evaluated.

**Methods:**

Fifty–two patients with obesity and 38 volunteers aged 20-50 years with normal body mass index (BMI), were enrolled in this cross-sectional study. Patients with diabetes, history of thyroid disorders, and patients, who were taking medications that influence their blood glucose or insulin levels or modified thyroid function tests, were excluded. TSH, free t4, insulin and glucose and thyroid sonography were carried out and the results compared between two groups. P<0.05 was considered as significant.

**Results:**

Thyroid volume was higher (p<0.001) and free t4 was lower (p<0.001) in patients with obesity but there was no difference in TSH between groups. Prevalence of thyroid nodules was 15.7% and 10.8% in obese and non-obese groups, respectively (p=0.51). Frequency of nodules was significantly higher in insulin resistant than non- insulin resistant subjects (22% vs.2%, p=0.01). BMI was associated with thyroid volume (r=0.44, p<0.001) and free t4 (r=-0.35, p=0.001). HOMA-IR (homeostatic model assessment for insulin resistance) had no correlation with thyroid volume (p=0.38), but associated with free t4 (r=-0.25, p=0.01).

**Conclusion:**

Free T4 was lower and volume of thyroid was higher in obese subjects, but TSH and frequency of thyroid nodules had no significant difference between obese and non-obese counterparts. Insulin resistant individuals had more nodules but thyroid volume was mainly associated with BMI.

Obesity is a chronic and prevalent health issue that is often associated with insulin resistance and hyperinsulinemia (1, 2). It seems that thyroid axis is influenced by obesity. But, there is uncertainty about the relationships between obesity and thyroid function. Both insulin resistance and visceral fat mass are considered as important factors in the relationship between obesity and thyroid function (3). A considerable amount of literature has been published on relationship between body mass index (BMI) and insulin resistance status with thyroid volume, its nodular disease and also thyroid hormones. But the results of these investigations have been different and occasionally contradictory (4-8). So, this study aims to contribute to this controversial area of the research by comparing the hormonal and anatomical characteristics of thyroid gland between obese and non-obese individuals. Also, as the second aim was to assess the relationship between various parameters of thyroid and insulin resistance status.

## Methods

In this cross-sectional study that was performed from November 2014 to February 2016, 52 participants with obesity (BMI ≥30) and 38 healthy volunteers with BMI<25, aged 20-50 years were enrolled. Of all the participants, 23.3% were males and 76.6% were females. Patients with diabetes, history of preceding or existing thyroid disorders, and patients who were taking medications that influence their blood glucose or insulin levels or modified thyroid function tests, were excluded.All participants signed an informed consent form, the study was approved by Ethicals Committee of Mashhad University of Medical Sciences (code: IR.MUMS.REC.1394.266).After an 8-hour fasting, blood samples were taken to measure the levels of plasma glucose, insulin, TSH and free t4. TSH was measured by IRMA (Sninjin Co, Korea) and free t4 by immunoassay (ADVIA Centaur CP). Reference range for TSH was 0.3-5 µu/ml and for free T4 was 0.8-1.7 ng/dl in women and 0.89-1.76 ng/dl in men. Fasting glucose levels were measured by glucose oxidase method (Pars Azmon Co, Iran) and insulin levels with IRMA (The Institute of Isotopes Co.Ltd, Budapest). Reference range for insulin was 5-25 µu/ml.HOMA-IR (Homeostatic Model Assessment for Insulin Resistance) calculated by this formula: FBS (mmol/L) × insulin (µu/ml)/22.5. While the subjects were minimally clothed and without shoes, weight and height were measured, also body mass index (BMI) was calculated by dividing weight (kg) by squared meters of height (m^2^). Thyroid sonography was carried out by the collaborative radiologist, using a high frequency linear probe (Samsung MedisonSonoAce x8). Volume of each lobe was calculated by this formula: length × width × thickness×0.52, the total volume of thyroid was determined by mean of volume of two lobes. Each discrete solid or solid–cystic thyroid lesion more than 3 mm was considered as thyroid nodule.

**Statistical analysis**: Statistical analysis was performed using SPSS software version 16.0.Chicago, SPSS Inc. The categorical variables were compared by chi-square or Fisher^,^s exact tests and Mann-Whitney U or Student t tests were used to compare the mean of variables between groups. The correlationbetween two variables was assessed by Spearman Rho or Pearson correlation tests, based on the distribution of variables. Total volume of thyroid that was not normally distributed was log-transformed for multiple linear regression analysis. A pvalue less than 0.05 was considered statistically significant.

## Results

In total, ninety participants were included in this study (52 with BMI ≥ 30 and 38 with BMI< 25), in group with obesity, 73.1% and in control group 81.6% of subjects were women, while 26.9% and 18.4% were men, respectively. There was no statistically significant difference between two groups according to gender. (p=0.346). 

Total volume of thyroid was significantly higher in males than females (6.04±2.99 vs. 4.50±2.33, p=0.013). Frequency of nodules was 15.7 % in subjects with obesity and 10.8% in normal BMI group but their difference was not statistically significant (p=0.51). When two groups were stratified based on sex, there was no significant difference in the presence of thyroid nodules between males (p=1.0) and also between female (p= 0.72) in two groups. 

**Figure1:**
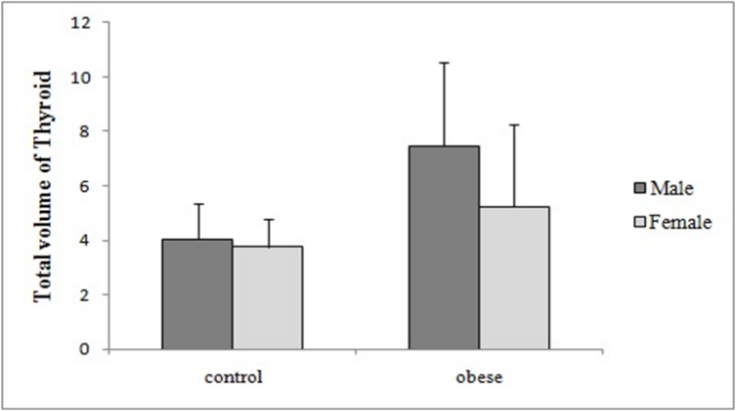
Comparison of thyroid volume between obese subjects and controls according to gender

**Figure 2:**
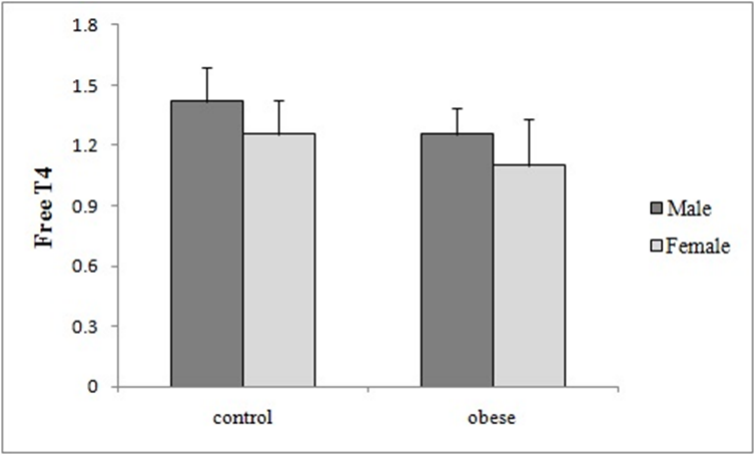
Comparison of free T4 between obese subjects and controls according to gender

Total volume of thyroid, fasting insulin and glucose, HOMA-IR, BMI and also age were all statistically significantly higher in obese group compared to control subjects, however the mean of freeT4 was significantly lower in obese group in comparison with control group (table 1). Comparison of thyroid volume and free T4 between patients with obesity and controls according to gender are shown in (table-2) and (fig.1) and (fig. 2), respectively.

**Table 1.** Comparisons of anthropometeric and laboratory findings between persons with obesity and normal BMI controls (Mean±SD)

variables	Control group (BMI<25)	Persons with obesity (BMI≥30)	Pvalue
**Total volume of thyroid **	3.81±1.04	5.81±3.13	<0.001
**Free T4**	1.28±0.17	1.14±0.21	<0.001
**TSH**	2.64±1.21	2.61±2.00	0.346
**insulin**	13.35±9.37	22.12±13.8	0.002
**Fasting blood suger**	79.72±10.58	99.94±18.58	<0.001
HOMA-IR**	2.74±2.36	5.75±4.14	<0.001
**Weight **	59.51±6.82	120.66±33.58	<0.001
**Height(meter)**	1.65±0.07	1.64±0.09	0.212
**BMI(kg/m** ^2^ **)†**	21.61±1.86	44.49±1.14	<0.001
**Age(year) **	26.74±8.50	39.45±7.92	<0.001

*All comparisons were done by Mann-Whitney test except for age,** HOMA-IR (Homeostatic Model Assessment for Insulin Resistance),† BMI(Body Mass Index)

**Table 2:** Comparison of thyroid volume and Free T4 between patients with obesity and controls according to gender (Mean±SD)

variable	gender	Patients with obesity	controls	P value
**Thyroid volume**	male	7.45±3.08	4.03±1.29	0.015
	female	5.24±2.99	3.76±0.99	0.016
**Free T4**	male	1.25±0.13	1.41±0.17	0.037
	female	1.10±0.22	1.25±0.16	0.003

Statistical analysis in all participants showed that the mean of HOMA-IR in group with thyroid nodules was significantly higher than group without nodules (p=0.01). HOMA-IR ≥2.5 was determined to confirm the presence of insulin resistance, where in the current study the thyroid nodules were significantly more prevalent in insulin resistant subjects than the non-insulin resistant ones (22% vs. 2% p=0.01). By evaluating the correlations, it was shown that in all participants, there was no correlation between BMI and TSH (p=0.98) and between thyroid volume and TSH (p=0.21). Similarly, the correlation between the total volume of thyroid and HOMA-IR was not significant (p=0.38). However, there was a significant positive correlation between the thyroid volume and BMI (p<0.001, r=0.44) and also between the thyroid volume and age (p=0.003, r= 0.34), respectively. Free t4 had a significant negative correlation with BMI (p=0.001, r=-0.35) and also with HOMA-IR (p=0.01, r=-0.25), respectively. There was no correlation between free t4 and age (p=0.08) and free t4 and thyroid volume (p=0.92). Due to the significant difference between age in two groups, and the presence of positive correlation between the thyroid volume and age and also the thyroid volume and BMI, we conducted a multiple linear regression with log of thyroid volume as a dependent variable and age and BMI as independent variables. Results showed that the coefficient of age was not significant (p=0.19).

## Discussion

In the current study, a significantly higher volume of thyroid and lower levels of free t4 in subjects with obesity were found. However, there was no difference in TSH between obese and non-obese counterparts. Similarly, there was no difference in the prevalence of thyroid nodules between the two groups. Furthermore, when all the participants were categorized into two groups with and without insulin resistance, the prevalence of thyroid nodules was significantly higher in insulin resistant group compared to non-insulin resistant group. Notably, a significant positive correlation was found between the thyroid volume and BMI, while there was no correlation between the thyroid volume and HOMA-IR. 

Finally, our study showed a significant negative correlation between free t4 and BMI and also the same association between free t4 and HOMA-IR in all participants. In support of current findings, Manji e al., illustrated no correlation between BMI and free t4 and also between BMI and TSH in obese patients with BMI ≥ 30 (4). Moreover, when the participants were categorized into two groups with BMI ≥ 30 and BMI< 30, they found no significant difference between median of TSH and also free t4 between two groups and no correlation was obtained between BMI and TSH and also BMI and free t4. According to a study conducted by Sousa et al., where they compared morbidly obese subjects (BMI≥40) with normal BMI group (BMI<25), no difference was derived in the prevalence of thyroid nodules between them, which was in accordance with our results. However, Sousa et al., showed a significant positive correlation between BMI and TSH and a significant positive association was detected between BMI and thyroid volume also thyroid volume with HOMA-IR, while there was no correlation between the thyroid volume and HOMA-IR in the current study. On the other hand, parallel to our findings, Sousa et al., found no correlation between TSH and thyroid volume (5).

 Makepeace et al. in a community-based, cross-sectional study showed a significant negative correlation between free t4 and BMI in never and former smokers, but not in current smokers (6). They found no positive correlation between TSH and BMI which was similar to current results. Knudsen et al., showed a significant negative correlation between free t4 and BMI after adjustment for smoking and also a significant positive association between BMI and TSH, this study was conducted in an iodine-deficient area (9), while in another study carried out by Nyrnes et al. in an iodine sufficient region and non-smokers showed a positive correlation between BMI and TSH (10). Therefore, it is possible that both iodine status and smoking be associated with thyroid function and weight changes (11, 12). In a study conducted by Marzullo et al., after controlling for confounders, there was no correlation between TSH and BMI, while there was a significant negative correlation between BMI and freeT4 and also between free t4 and HOMA-IR, these results were in accordance with our findings (7). The NHANES 2007-2008 survey in euthyroid subjects, showed a positive correlation between BMI and TSH, however, not between BMI and freeT4 (13). It is believed that, leptin level increases and adiponectin decreases in obese subjects and in normal individuals, there is a direct correlation between freeT4 and adiponectin (14-15). Therefore, one of the possible explanations for low freeT4 levels in obese subjects compared to normal subjects is the reduction in adiponectin levels which is suggested to be associated with increase in leptin levels. Also, increase in fat free mass during obesity is associated with increase in T4 disposal that leads to low/normal free t4 (16). Notably, increase in production of T3 from T4 by deiodinase 2 that increases resting energy expenditure might be another mechanism associated with lower freeT4 in subjects with obesity compared to normal subjects (17). According to two studies conducted by Iacobellis and Galofre et al., there was a positive correlation between insulin resistance and thyroid function in obese subjects (18-19).Also, it has been demonstrated that in diabetic patients with thyroid nodules, HOMA-IR is higher than diabetic patients without thyroid nodule (20). In accordance with Tang investigation (20). Heidari et al., illustrated that in individuals with normal glucose metabolism who had thyroid nodules, HOMA-IR was higher compared to subjects without thyroid nodules (21). Interestingly, when adjustment for BMI was done, the correlation between thyroid hormones and insulin resistance was not significant (22). Difference in the study design, definition of normal range of TSH and freeT4, degree of obesity (morbid or non-morbid), iodine and smoking status and other factors that are unknown at present may be the possible causes for disparity in the current findings. 

One of the limitations of our study was the lack of evaluation on smoking status in both study groups. Another limitation of current study was the cross-sectional design of study that could not explain casualty. Therefore, our findings need to be evaluated in prospective cohort studies. Moreover, T3 or freeT3 were not measured in the current study, thus we cannot discuss the possible mechanism and relationship between T3 and free t4. Future studies with appropriate designs and without limitations mentioned above will help us to better demonstrate the relationship between thyroid status and obesity. 

In conclusion free t4 was lower and volume of thyroid was higher in obese subjects, but TSH and frequency of thyroid nodules had no significant difference between obese and non-obese counterparts. Insulin resistant individuals had more nodules but thyroid volume was mainly associated with BMI. These relationships should be examined further in future clinical studies.
